# Predictive Potential of Twenty-Two Biochemical Biomarkers for Coronary Artery Disease in Type 2 Diabetes Mellitus

**DOI:** 10.1155/2015/146816

**Published:** 2015-05-18

**Authors:** Edimar Cristiano Pereira, Marcelo Chiara Bertolami, André Arpad Faludi, Osmar Monte, Hermes Toros Xavier, Tiago Veiga Pereira, Dulcineia Saes Parra Abdalla

**Affiliations:** ^1^Faculdade de Ciências Farmacêuticas, Universidade de São Paulo, 05508-900 São Paulo, SP, Brazil; ^2^Universidade Federal de São Paulo, 09913-030 Diadema, SP, Brazil; ^3^Instituto Dante Pazzanese de Cardiologia, 04012-180 São Paulo, SP, Brazil; ^4^Faculdade de Ciências M'edicas, Universidade Metodista de Santos, 11045-101 Santos, SP, Brazil; ^5^Santa Casa de Misericórdia de São Paulo, 01221-020 São Paulo, SP, Brazil Santa Casa de Misericórdia de São Paulo, 01221-020 São Paulo, SP, Brazil; ^6^Unidade de Avaliação de Tecnologias em Saúde, Instituto de Educação e Ciências em Saúde, Hospital Alemão Oswaldo Cruz, 01323-903 São Paulo, SP, Brazil; ^7^Laboratório de Genética e Cardiologia Molecular, Instituto do Coração (InCor), 05403-900 São Paulo, SP, Brazil

## Abstract

We investigated the potential of a panel of 22 biomarkers to predict the presence of coronary artery disease (CAD) in type 2 diabetes mellitus (DM2) patients. The study enrolled 96 DM2 patients with (*n* = 75) and without (*n* = 21) evidence of CAD. We assessed a biochemical profile that included 22 biomarkers: total cholesterol, LDL, HDL, LDL/HDL, triglycerides, glucose, glycated hemoglobin, fructosamine, homocysteine, cysteine, methionine, reduced glutathione, oxidized glutathione, reduced glutathione/oxidized glutathione, L-arginine, asymmetric dimethyl-L-arginine, symmetric dimethyl-L-arginine, asymmetric dimethyl-L-arginine/L-arginine, nitrate plus nitrite, S-nitrosothiols, nitrotyrosine, and n-acetyl-*β*-glucosaminidase. Prediction models were built using logistic regression models. We found that eight biomarkers (methionine, nitratate plus nitrite, n-acetyl-*β*-glucosaminidase, BMI, LDL, HDL, reduced glutathione, and L-arginine/asymmetric dimethyl-L-arginine) along with gender and BMI were significantly associated with the odds of CAD in DM2. These preliminary findings support the notion that emerging biochemical markers might be used for CAD prediction in patients with DM2. Our findings warrant further investigation with large, well-designed studies.

## 1. Introduction

Type 2 diabetes mellitus (DM2) is an important risk factor for atherosclerosis. Chronic hyperglycemia is related to pathophysiology of macrovascular and microvascular diseases. Even small changes in glucose metabolism may contribute to the onset of cardiovascular disease and endothelial dysfunction due to peripheral insulin resistance. Acute myocardial infarction affects a large proportion of patients with DM2; thus, the evidence of subclinical atherosclerosis would be helpful for preventive strategies in these individuals [1, 2].

Even knowing that hyperglycemia is one of the ways that can lead to the development of atherosclerosis, the mechanisms underlying coronary artery disease (CAD) in individuals with type II diabetes are not known for sure. Thus, besides serum glucose, other markers related to the pathophysiology of cardiovascular diseases might also be evaluated. Recent prominent candidates for prediction of CAD in DM2 patients include thiols (cysteine, homocysteine, methionine oxidized glutathione, and reduced glutathione), N-acetyl-*β*-D-glucosaminidase (NAGase), endogenous nitric oxide synthase inhibitors (ADMA), nitrate + nitrite (NOx), nitrotyrosine, and S-nitrosothiols (RSNO). Hence, the purpose of this study was to identify important biochemical alterations that could distinguish DM2 individuals with and without CAD.

## 2. Materials and Methods

### 2.1. Study Participants

In this study, 96 volunteers were enrolled and classified into the following groups: type 2 diabetes mellitus (DM2, *n* = 21) and type 2 diabetes mellitus with coronary artery disease (DM2 + CAD, *n* = 75) (Table 1). Volunteers of both genders (aged ≥ 21 years) were screened at the following Brazilian institutions: Instituto Dante Pazzanese de Cardiologia, Irmandade da Santa Casa de Misericórdia de São Paulo, and Faculdade de Ciências Médicas da Universidade Metodista de Santos. All subjects were fully informed about the details of the study and protocol and provided written informed consent. The present study was approved by the ethics committees of the three participating institutions. DM2 was defined according to the American Diabetes Society criteria, whereas participants with coronary artery disease were defined as patients who had acute myocardial infarctions confirmed by ECG, laboratory exams, and clinical symptoms [3]. Exclusion criteria used were pregnancy, psychiatric disorders, renal and/or hepatic diseases, smoking, alcoholism, cancer, or other pathological conditions that could interfere with the study. Venous blood was collected in tubes with and without EDTA after 12 hours of fasting. Serum and plasma were obtained by centrifugation and stored frozen at −70°C.

### 2.2. Biochemical Analysis

The concentrations of fructosamine, glycated hemoglobin (HbA_1C_), glucose, total cholesterol, triglyceride, high-density lipoprotein- (HDL-) cholesterol, and very low density lipoprotein- (VLDL-) cholesterol were estimated by enzymatic methods using kits (Biosystems SA, Barcelona, Spain). The Friedewald equation was used to calculate the low density lipoprotein- (LDL-) cholesterol.

### 2.3. N-Acetyl-*β*-glucosaminidase (NAGase) Activity

N-Acetyl-*β*-glucosaminidase activity was determined based on the methodology described by Reglero and Cabezas (1976). 100 *μ*L of plasma was added to 1.25 mL citrate buffer (0.1 M, pH 4.4) and incubated at 37°C with 0.25 mL 0.01 M p-nitrophenyl-N-acetyl-glucosaminide in citrate buffer for 15 minutes. The reaction was interrupted with 1.5 mL of sodium carbonate (0.2 M, pH 10.4) and the final product was measured by spectrophotometer at 405 nm. The calibration curve was made using standard solution of p-nitrophenol. The NAGase activity of the samples was calculated after subtraction of the blank sample. One unit of enzyme was defined as the amount released 1 *μ*mol p-nitrophenol/min [4].

### 2.4. NOx (Nitrate + Nitrite)

We used the nitric oxide analyzer (NOA^280^, Sievers, USA) based on the chemiluminescence reaction between nitric oxide and ozone. The reduction of nitrate and nitrite (NOx) with vanadium chloride was used to convert NOx to oxide nitric. Reduction was done at 90°C in 0.1 M HCl. Calibration curves with multiple levels were performed with an external standard (sodium nitrate) using the Bag Program software (version 2.2, Sievers, USA). The samples were extracted with cold ethanol (0.5 mL sample and 1.0 mL of ethanol at 0°C). After vortexing, the solution was stored on ice during 30 minutes and then centrifuged at 9000 g for fifteen minutes. The supernatant was removed and analyzed in nitric oxide analyzer [5].

### 2.5. S-Nitrosothiols

The standard S-nitroso-albumin (SNO-Alb) was used to quantify total S-ntrosothiol serum. The synthesis was obtained by the reaction of nitrite with human albumin in 0.1 M HCl. This was incubated in the dark for 2 hours and then the absorbance was measured at 336 mm (*ε* = 3874 M^−1 ^cm^−1^). The calibration curve was made with 10, 50, 100, 250, and 500 nM SNO-Alb. 1.0 mL of plasma was added to 10 *μ*L of n-ethylmaleimide (500 mM). After homogenizing, 100 *μ*L of sulfanilamide (1%) was added and homogenized again. Then the samples were stored on ice until analysis. We used the equipment Nitric Oxide Analyzer (NOA^280^, Sievers, USA) and injected 500 *μ*L of sample and 500 *μ*L of standard. The reaction solution was composed of 8 mL of glacial acetic acid, 2 mL of KI (50 mg/mL), 300 *μ*L of decanol, and 200 *μ*L of CuSO_4_ (200 mM) at 70°C [6].

### 2.6. ADMA (Asymmetric Dimethyl-L-arginine), SDMA (Symmetric Dimethyl-L-arginine), and L-Arginine

Plasma concentrations of ADMA, SDMA, and L-arginine were determined by the technique of capillary electrophoresis (BioFocus 2000, Bio-Rad Laboratories, Inc.). The blood was collected with EDTA and centrifuged at 1,000 g for 10 minutes at 4°C to obtain plasma. It was added to the plasma L-homoarginine as an internal standard. The calibration curve was performed by adding the standard of ADMA, SDMA, and L-arginine in pooled plasma. Samples and standard were precipitated with ethanol, centrifuged at 9.000 g for 15 minutes at 4°C, and then derivatized with fluorescein 5-isothiocyanate. The injection was done under pressure (1 psi.sec) and the race was performed in a fused silica capillary (85 cm length and 50 internal diameter *μ*m) at 20 kV. Running buffer consisted of 50 mM boric acid and 20 mM 3-cyclohexylamino-1-propanesulfonic adjusted to pH 10.8. The detector laser-induced fluorescence (BioFocus LIF^2^, Bio-Rad Laboratories, Inc.) operated at 488 nm (excitation) and 520 nm (emission) (Caussé et al. 2000) [7].

### 2.7. Thiols (Homocysteine, Cysteine, Methionine, Oxidized Glutathione, and Reduced Glutathione)

The blood was collected with EDTA and centrifuged at 1000 g for 10 minutes at 4°C to obtain plasma. In 200 *μ*L in plasma 2 *μ*L internal standard (1 mM n-(2-mercaptopropionyl) glycine) and 20 *μ*L tri-n-butylphosphine 10% (v/v dimethylformamide) were added. The preparation was homogenized and incubated for 30 minutes at 4°C. Then, 200 *μ*L 10% trichloroacetic acid containing 1 mM EDTA was added. After homogenizing samples, they were centrifuged at 13.000 g for 15 minutes at 4°C. In 100 *μ*L of the supernatant was added to 100 *μ*L 0.5 M phosphate buffer and adjusted to pH 7.5 with 1 M Na_3_PO_4_. The derivatizer (5-bromomethylfluorescein ) was added to the samples at a molar ratio of 5 to 10 times excess and the preparations are incubated for 15 minutes at 60°C. After derivatization, the sample was diluted at 1 : 10 with phosphate buffer 0.25 M pH 7.6 and injected with a pressure of 0.5 psi for 1.5 seconds on capillary (70 cm length and 50 *μ*m internal diameter). The capillary electrophoresis instrument (BioFocus 2000, Bio-Rad Laboratories, Inc.) was performed at 25°C, 30 kV, and positive polarity on injection. The detector laser-induced fluorescence (BioFocus LIF^2^, Bio-Rad Laboratories, Inc.) operated in 488 nm (excitation) and 520 nm (emission). For each run the capillary was washed with 1 M NaOH (Vecchione et al. 1999) [8].

### 2.8. Nitrotyrosine

The nitrotyrosine in proteins was determined by a competitive ELISA method. We used the polyclonal anti-nitrotyrosine antibody (Upstate, Catalog Number: 06-284). The standard used was nitrated bovine albumin (nitro-albumin) prepared by alkaline addition of 1 mM peroxynitrite and 1 mM albumin. The concentration of the nitro-albumin was determined using the molar extinction coefficient of 4300 m^−1 ^cm^−1^ at 438 nm and pH 9.0. The plate was sensitized with 0.05 ug nitro-albumin per well and then washed, blocked with milk protein, and rinsed again. The following was added to the plate anti-nitrotyrosine antibody and sample/standard. After incubation, the plate was washed and added to the peroxidase-conjugated antibody (Stressgen Biotechnologies Corp.). After incubation and washing, 2.3 mM luminol and 0.9 mM p-iodophenol (200 uL/well) and 3.9 mM hydrogen peroxide (50 uL/well) were added. The reading of the chemiluminescence produced was immediately performed (LumiCount, Packard, Meriden, USA). The present nitrotyrosine concentrations in the sample were estimated by using the calibration curve and nitrated albumin was expressed as equivalent nitro-albumin [9].

### 2.9. Statistical Analysis

There was no missing data in our study. Data were expressed as means ± standard deviation (SD), median (interquartile range), or counts (percentage) when appropriate. For univariate analyses, groups were compared by the *t*-test for approximately normally distributed variables or by means of the Mann-Whitney* U*-test for variables with skewed distribution. Fisher's exact test was used to test differences in count data. In addition, we constructed multiple logistic regression models using the backward stepwise selection procedure to ascertain predictors for CAD in DM2 patients (coding scheme: DM2 + CAD = 1, DM2 = 0). Variables were sequentially removed from the full model (all regression terms included) when the correspondent *P* value was higher than 0.10. In order to assess the model performance, we did not employ* k*-fold partitioning due to the relatively low number of subjects. Instead, we used the resubstitution approach, in which the same data are used for both training and testing [10]. For these analyses, each participant has an estimated probability of being DM2 + CAD, which is calculated from the logistic model-derived equation based on the participant's own variables. If the calculated probability was ≥50%, the subject was assigned as DM2 + CAD or DM2 only, otherwise. Model predictions were then compared to the true (known) status. We quantified model performance by using two measures of classification accuracy that are not susceptible to class imbalance: balanced accuracy (BA) and normalized mutual information (NMI).

BA is an accuracy measure that takes into account both sensitivity and specificity of the models and is calculated as the average of sensitivity and specificity. This accuracy measure ranges from 0 to 100%. NMI is an information-theoretic measure of classifier performance. NMI ranges from 0 to 100% and is interpreted as the amount by which the examined model reduces one's uncertainty about the true state of the participants (e.g., 0% means that the status is independent of the studied explanatory variables, while 100% suggests that the model fully predicts the status for each individual) [11]. Permutation was used to construct empirical null distributions (2,000 shuffles) in order to compute the statistical significance of both BA and MNI measures. The hypothesis of a significantly better fit for the model with biochemical biomarkers plus classical variables for CAD (full model) compared to the simpler models (*nested* models) was tested by means of a likelihood-ratio test. This test assumes the form Δ = −2(*l*_0_ − *l*_1_), where *l*_1_ and *l*_0_ are the natural log-likelihoods under the full and nested models, respectively. We used permutation to test whether the full model fits the data significantly better than simpler models by comparing the observed test statistic Δ to those obtained in 2,000 randomly generated shuffles. *P* values were adjusted by the Holm-Bonferroni procedure in sensitivity analyses [11]. All data analyses were performed using the Stata package (version 11.0, STATA Corp., College Station, TX, USA). Two-sided *P* values < .05 were considered statistically significant.

## 3. Results

### 3.1. Analytical Validation

All methodologies presented in this study showed satisfactory accuracy and replication. All calibration curves were linear with a coefficient of determination (*R* ^2^) ≥ 0.98. The quantification limit (10 : 1; signal : noise) by capillary electrophoresis (ADMA, SDMA, and L-arginine: 25 nM; thiols: 50 nM) and nitric oxide analyzer (NOx and RSNO: 10 nM) were suitable for detection of the analytes in the samples. The ELISA also showed a quantification limit adequate for detection of nitrotyrosine (30 nM). All samples and standards were analyzed in duplicate or triplicate.

### 3.2. Participants' Biochemical and Demographic Characteristics

The main demographical, clinical, and biochemical characteristics of the studied participants are shown in Table 1. Twenty-one (22%) out of 96 participants with DM2 were classified as DM2 + CAD. Age, body mass index (BMI), and gender proportion were comparable between DM2 + CAD and DM2 groups. However, in an exploratory (unadjusted) analysis, levels of methionine, LDL/HDL ratio, N-acetyl-*β*-glucosaminidase, and nitrite plus nitrate (NOx) were significantly higher in DM2 + CAD. In addition, levels of L-arginine : ADMA ratio, fructosamine, and reduced glutathione were significantly lower in DM2 + CAD patients compared to their DM2 counterparts. After a Holm-Bonferroni correction for 25 tests, both NOx and fructosamine levels remained significantly higher in DM2 + CAD participants compared to the DM2 group (*P* = 0.02 and *P* = 0.006, resp.).

### 3.3. Predicting CAD in DM2

To determine putative predictor variables for CAD in DM2, we built a multiple logistic regression model with backward elimination. Of the 25 variables tested, eight remained in the final model: LDL/HDL ratio, methionine, reduced glutathione (GSH), L-arginine : ADMA, NOx (nitrate plus nitrite), N-acetyl-*β*-glucosaminidase (NAGase), gender, and BMI (Table 2). On the basis of this model (hereafter named* full model*), we constructed an equation that was applied to the complete dataset to predict the status of the 96 studied participants. The overall classification accuracy of this model (resubstitution method) is presented in Table 3, along with other two competing models including either only classical variables for CAD (i.e., LDL/HDL ratio, gender, and BMI; model 2) or emerging biochemical markers only (i.e., L-arginine : ADMA ratio, methionine, GSH, NAGase, and NOx; model 3).

All of the three models significantly predicted CAD more often than can be expected by chance, although it can be verified from Table 3 that the performance of the studied models ranges from small to moderate.

### 3.4. The Added Value of Emerging Biochemical Markers for CAD Prediction

We sought next to assess whether there is an increase in accuracy for prediction models when emerging biochemical markers are used in combination with classical risk factors for CAD. In other words, we tested whether simpler models (models 2 and 3) are equally capable of predicting CAD in DM2 patients compared to the full model. This is of paramount importance, since there is a potential of overfitting as the number of variables included in the model increases.

We observed that the full model fits significantly better the data compared to either model 2 (*P* < 0.001) or model 3 (*P* = 0.004), indicating that there is a statistically significant gain in accuracy when both emerging biochemical markers and classical risk factors are added to the model for CAD prediction.

For comparison purposes, less conservative measures of diagnostic accuracy (e.g., classification error) also favor the full model over models 2 and 3 (data not shown).

## 4. Discussion

### 4.1. Main Findings

We found that individuals with DM2 + CAD have several significant metabolic changes related to endothelial dysfunction and oxidative stress compared to DM2 without CAD. In our sample, we observed significant alterations in the following variables: HDL-cholesterol, fructosamine, GSH, SDMA, L-arginine/ADMA, NOx, and NAGase.

In addition, we showed that the prediction accuracy for CAD in DM2 patients is significantly increased when six emerging biochemical markers are incorporated into the model along with classical risk factors for CAD.

### 4.2. Study Limitations

Our study has a number of important limitations. First, our investigation should be regarded as a “predictor finding study” only. Although we have developed a multivariable prediction model, we did not aim at the development of a model for use in clinical practice to guide patient management [12]. Instead, our study should be viewed as a hypothesis-generating study that warrants further confirmation in larger, well-powered investigations with external validation. Second, criticism might be directed at the fact that our definition of CAD might lack specificity. Because CAD was classified conservatively, silent CAD might be present among a few patients in the DM2 + CAD group. However, in our study, misclassification of the CAD status is likely to lead to downwardly biased estimates, ultimately leading to less impressive model predictions (i.e., more conservative models). Third, our sampling scheme was based on volunteers and was not designed to be representative of the population. With that said, our results are prone to a survival bias, since there is a reduced likelihood of enrolling patients who died acutely or who are experiencing severe consequences of myocardial infarction [13]. Again, these results are likely to lead to more conservative model predictions, since more prominent metabolic alterations are plausible in advanced CAD patients. On the other hand, even though we employed permutation to compute statistical significance, our small sample size is prone to overoptimistic results [14]. Indeed, there is a clear potential for overfitting and overestimation, because our models were constructed with a large number of biomarkers relative to the number of participants [15].

### 4.3. Usefulness of Novel Biochemical Markers for CAD Prediction in DM2

In another study conducted by our research group [16] we showed that hyperglycemia correlated with increased production of nitric oxide based on the evaluation of concentrations of nitrate and nitrite. Endothelial dysfunction is generally defined as impaired endothelium-dependent vasodilation, related to lower ^•^NO production or bioavailability. Reactive oxygen and nitrogen species (RONS), such as H_2_O_2_, O_2_^•−^, ^•^NO, and ONOO^−^, can be produced in blood vessels by certain drugs and pathological conditions and accentuate or induce endothelial dysfunction [17]. RONS may reduce ^•^NO bioactivity by formation of peroxynitrite or by decrease of enzyme or transporter activities by oxidation of their thiols groups [18]. Endothelial dysfunction can contribute to the initiation, progression, and clinical manifestations of CAD. The increase in S-nitrosothiols may be a consequence of glycation of proteins, which facilitates the nitrosation of protein thiols by nitric oxide [19]. The glycated proteins can act as a sink for nitric oxide because S-nitrosation reduces their competence to release nitric oxide which reflects directly on the pathogenesis of endothelial dysfunction [20]. Nitric oxide can react with superoxide anion, which leads to the formation of a potent oxidant, peroxynitrite, and in sequence generation of hydroxyl radicals and nitrogen dioxide [18, 21, 22]. The tyrosine, free or protein-bind, may react with nitrogen dioxide generating nitrotyrosine, which is considered a biomarker of the formation of peroxynitrite [23]. In this present study, only the increase of NOx was demonstrated with no differences for nitrotyrosine, even though reduced glutathione and reduced glutathione/oxidized glutathione ratio concentrations were observed.

The endogenous nitric oxide synthase inhibitors, represented by symmetric dimethylarginine (SDMA) and asymmetric dimethylarginine (ADMA), also play an important role in endothelial dysfunction. In the present study, reduced concentration of L-arginine/ADMA in DM2 + CAD group in comparison with DM2 without CAD reinforces the fact that endothelial nitric oxide synthase inhibition occurs along the atherosclerosis process. Accordingly, it has been shown that L-arginine/ADMA levels diminished parallel to the impairment of vasodilatation in DM2 patients after lipid ingestion [24]. Moreover, it was previously reported that low L-arginine/ADMA ratio was not related to either the presence of macroangiopathy or the time of disease manifestation in DM2 patients, but rather to the metabolic control of the diabetes [25]. Thus, in the present study, hypercholesterolemia observed in DM2 + CAD group could be related to ADMA increase, occasioning a lower production of  ^•^NO and leading to endothelial dysfunction. However, nitric oxide low production could not be confirmed in this study based only on the increase of nitrite plus nitrate concentration generation. Studies have shown a reduction of endothelial dysfunction by rising L-arginine/ADMA ratio [26]. The source of ADMA in hypercholesterolemia is unknown. ADMA can be the result of the hydrolysis of methylated proteins [27]. In vivo lipid peroxidation causes peroxidative damage to tissue proteins and may raise the rate of proteolysis. Alternatively, there may be a dysfunction or downregulation of di-methylarginine dimethylaminohydrolase, the enzyme that converts ADMA to L-citrulline [28]. Hypercholesterolemia may alter the regulation or function of di-methylarginine dimethylaminohydrolase, thereby resulting in intracellular accumulation of ADMA. In fact, regenerating endothelial cells impairs vasodilation and produces more ADMA [29]. Hypercholesterolemia plus hyperglycemia is a factor that contributes to oxidative stress due to autoxidation of glucose and nonenzymatic protein glycation.

Thiols play an important role on the oxidative processes and therefore may directly or indirectly contribute in endothelial dysfunction. Glutathione is a major thiol used as a biomarker of oxidative stress and is found in the forms of GSH (reduced glutathione) and GSSG (oxidized glutathione). In the present study, the DM2 + CAD group showed lower GSH/GSSG and GSH concentration, compared with DM2 without CAD group. This decrease of reduced glutathione may be due to activation of the polyol pathway in which glucose is reduced to sorbitol by the NADPH-dependent aldose reductase [30, 31]. This enzyme presents a high Michaelis-Menten constant for glucose and therefore this metabolic via is only quantitatively significant in hyperglycemia. Thus, whenever the NADPH/NADP ratio is decreased in hyperglycemic state, it will result in prejudice in the regeneration of reduced glutathione. Also as a consequence, the synthesis of nitric oxide by nitric oxide synthase will be impaired due to the NADPH dependence [31, 32]. Apparently, the decrease in GSH concentration has a relation to the presence of atherosclerotic inflammation process in patients with CAD.

The activity of N-acetyl-*β*-D-glucosaminidase (NAGase) in plasma has been used as a biomarker of endothelial dysfunction [33]. In the present study, NAGase activity was significantly higher in DM2 + CAD group in comparison with the DM2 without CAD group. Previous studies have reported increased NAGase activity in individuals with CAD [34–36]. NAGase is a lysosomal enzyme produced by many cells, including not only endothelial cells, but also smooth muscle and kidney [37]. These enzymes can be thrown into the extracellular environment through stimulation of oxidative stress. Sánchez-Hueso et al. showed correlation between measurements of brachial artery diameter and blood flow via Doppler ultrasonography with NAGase activity in patients with DAC [35]. Additionally, NAGase activity showed positively correlated with insulin resistance in patients with CAD [38]. According to Komosińska-Vassev et al., the increase of NAGase activity can be considered an indicator of the intensity of endothelial cell dysfunction [39]. Several studies also demonstrated that NAGase is increased in individuals with DM2, when compared to healthy individuals and that NAGase activity is high in individuals with DM2 and previous history of cardiovascular disease (myocardial infarction) in comparison with individuals with DM2 without previous coronary events [34, 35, 37]. Thus, our data reinforce previous data showing that the increase of NAGase activity in individuals with DM2 can be associated with the presence of CAD.

## Figures and Tables

**Table 1:** The demographical clinical and biochemical characteristics of the 96 patients.

	DM2 + CAD (*n* = 75)	DM2 (*n* = 21)	*P*
Age (years)	59 (57–62)	59 (52–64)	0.66
BMI (kg/m^2^)	29.1 ± 2.90	28.0 ± 2.96	0.11
Cholesterol (mg/dL)	216.7 ± 50.6	208.5 ± 41.1	0.53
Gender			
Male	10 (48%)	32 (43%)	0.80
Female	11 (52%)	43 (57%)	
LDL-cholesterol (mg/dL)	129 (111–146)	124 (98–164)	0.92
HDL-cholesterol (mg/dL)	35 (28–47)	45 (35–54)	0.01
Triglycerides (mg/dL)	185 (132–216)	184 (131–253)	0.60
LDL-cholesterol/HDL-cholesterol	3.23 (2.60–5.18)	2.88 (2.21–3.84)	0.08
Glucose (mg/dL)	164.1 ± 59.1	169.6 ± 88.3	0.48
Glycated hemoglobin (%)	8.4 (7.2–10.1)	8.2 (6.9–9.6)	0.69
Fructosamine (mg/dL)	2.96 (2.43–3.18)	3.83 (2.99–6.47)	<0.001
Homocysteine (*µ*mol/L)	6.5 (3.5–10.6)	8.5 (5.6–12.3)	0.21
Cysteine (*µ*mol/L)	168 (149–217)	198 (158–245)	0.17
Methionine (*µ*mol/L)	21.2 (19.2–43.4)	26.0 (19.0–35.4)	0.81
Reduced glutathione (*µ*mol/L)	3.28 (2.47–4.55)	4.05 (3.17–5.54)	0.03
Oxidized glutathione (*µ*mol/L)	0.92 (0.68–1.16)	0.94 (0.66–1.34)	0.58
Reduced glutathione/oxidized glutathione	3.11 (2.69–4.47)	3.96 (3.10–6.56)	0.05
L-Arginine (*µ*mol/L)	48.4 (35.8–57.3)	49.3 (35.6–68.0)	0.37
Asymmetric dimethyl-L-arginine (*µ*mol/L)	0.96 (0.88–1.46)	0.89 (0.69–1.22)	0.08
Symmetric dimethyl-L-arginine (*µ*mol/L)	0.70 (0.49–0.82)	0.79 (0.62–1.03)	0.04
L-Arginine/asymmetric dimethyl-L-arginine	39.4 (35.9–43.7)	51.9 (35.3–70.9)	0.02
Nitrate plus nitrite (*µ*mol/L)	50.1 (36.9–57.0)	32.1 (25.5–41.1)	<0.001
S-Nitrosothiols (nM)	129 (93–164)	131 (95–170)	0.54
Nitrotyrosine (nM)	435 (375–453)	453 (315–615)	0.59
n-Acetyl-*β*-glucosaminidase (U/L)	33.2 (28.3–41.2)	26.3 (22.8–34.4)	0.02

DM2: type 2 diabetes mellitus. CAD: coronary artery disease. Results are given as means ± standard deviation, median (interquartile range), or counts (percentage). *P* values shown are not adjusted for multiple testing and refer to the univariate analysis.

**Table 2:** Final multiple logistic regression model for coronary artery disease in patients with type 2 diabetes (*n* = 96).

	Coefficient (*β*)	SE of *β*	*Z*	*P*	Odds ratio	95% CI
Intercept	−21.51	6.501	—	—	—	—
LDL-cholesterol/HDL-cholesterol	0.690	0.290	2.38	0.017	1.99	1.13 to 3.52
Methionine (*µ*mol/L)	0.083	0.031	2.68	0.007	1.09	1.02 to 1.16
Reduced glutathione (*µ*mol/L)	−0.512	0.261	−1.96	0.050	0.60	0.36 to 1.00
L-Arginine/asymmetric dimethyl-L-arginine	−0.052	0.022	−2.30	0.021	0.95	0.91 to 0.99
Nitrate plus nitrite (*µ*mol/L)	0.095	0.028	3.40	0.001	1.10	1.04 to 1.16
n-Acetyl-*β*-glucosaminidase (U/L)	0.087	0.038	2.27	0.023	1.09	1.01 to 1.17
Gender (male = 1, female = 0)	−2.667	1.033	−2.58	0.010	0.07	0.01 to 0.52
BMI (kg/m^2^)	0.588	0.189	3.11	0.002	1.80	1.24 to 2.61

(*β*): coefficient that reflects the expected increase in the log⁡(odds ratio) for CAD per unit increase in the independent variable. SE: standard error. CI: confidence intervals.

**Table 3:** Performance of different models for CAD prediction in type 2 diabetes patients.

	BA (%)	*P*_BA_^*^	NMI (%)	*P*_NMI_^*^
*Model 1* (full): classical factors + emerging biochemical factors	72.85%	0.002	20.52%	0.001
*Model 2*: classical factors only	57.14%	0.01	9.29%	0.003
*Model 3*: emerging biochemical factors only	66.38%	0.001	13.08%	0.003

BA: balanced accuracy. NMI: normalized mutual information.

*Model 1*: LDL: HDL ratio, methionine, reduced glutathione, L-arginine : asymmetric dimethyl-L-arginine, nitrate plus nitrite, n-acetyl-*β*-glucosaminidase, gender, and BMI.

*Model 2*: LDL : HDL ratio, gender, and BMI.

*Model 3*: methionine, reduced glutathione, L-arginine : asymmetric dimethyl-L-arginine, nitrate plus nitrite, and n-acetyl-*β*-glucosaminidase.

^*^ *P* value based on 2,000 permutations. Age was not a significant predictor, yielding to virtually identical results after inclusion of this variable in the models (data not shown).
